# *Giardia* unveiled: a proteome view of the parasite in search of drug, vaccine and diagnostic targets

**DOI:** 10.1017/S0031182026101899

**Published:** 2026-04

**Authors:** Lorena González-López, Oscar Rodríguez-Lima, Blanca Esther Blancas-Luciano, Margarita Jacaranda Rosendo-Pineda, Nancy Guadalupe Velázquez Zavala, Luis Vaca, Margarita Cabrera-Bravo, Adolfo Cruz-Reséndiz

**Affiliations:** 1 Departamento de Salud Pública, Facultad de Medicina, Universidad Nacional Autónoma de México, Ciudad de México, México; 2 Departamento de Microbiología y Parasitología, Facultad de Medicina, Universidad Nacional Autónoma de México, Ciudad de México, México; 3 Departamento de Biología Celular, Facultad de Ciencias, Universidad Nacional Autónoma de México, Ciudad de México, México; 4 Sección de Laboratorios, Subsección Anatomía, Escuela Militar de Medicina, Ciudad de México, México; 5 Departamento de Bioquímica y Biología Estructural, Instituto de Fisiología Celular, Universidad Nacional Autónoma de México, Ciudad de México, México; 6 Departamento de Biología Celular y Del Desarrollo, Instituto de Fisiología Celular, Universidad Nacional Autónoma de México, Ciudad de México, México

**Keywords:** diagnostic targets, drug targets, *Giardia lamblia*, proteome, vaccine targets

## Abstract

Giardiasis remains a significant global health burden, constrained by limited diagnostic tools, the emergence of drug-resistant *Giardia lamblia* strains, and the absence of a licenced human vaccine. To address these critical gaps, this review provides a comprehensive functional analysis of the *Giardia* proteome, emphasizing molecular targets essential for the parasite’s survival and pathogenesis. We systematically examine the structural proteome, specifically the tubulin reservoir and the diverse giardin family (α-, β-, γ- and δ-giardins), elucidating their indispensable roles in the ventral disc attachment mechanism. Beyond structural components, we detail the ‘pathoproteome’, and moonlighting enzymes, highlighting how the secretome – including cathepsin B-like cysteine proteases (notably giardipain-1) and variant-specific surface proteins facilitate immune evasion and host intestinal epithelial damage. Furthermore, the review explores the metabolic and encystation proteomes, identifying unique enzymes such as carbamate kinase and fructose 1,6-bisphosphate aldolase that offer high therapeutic selectivity. By synthesizing these proteomic insights, this work identifies high-priority candidates for the development of next-generation therapeutics, prophylactic, and diagnostic interventions aimed at mitigating the global impact of this neglected disease.

## Graphical abstract

**Figure:**
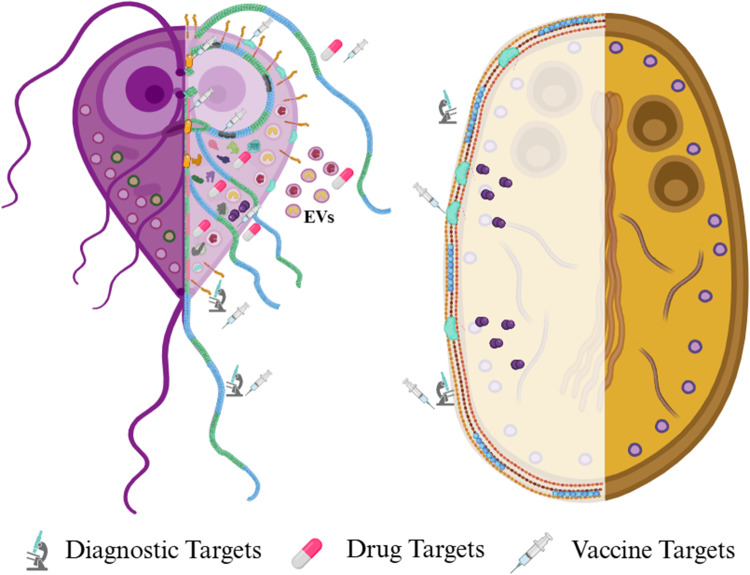

## Introduction

Giardiasis is a parasitic disease caused by the non-invasive, flagellated protozoan *Giardia lamblia* (syn. *G. duodenalis* or *G. intestinalis*), which colonizes the small intestine of humans and other mammals (McInally and Dawson, 2016; Dixon, 2021). *G. lamblia* is categorized into 8 genetic assemblages (A–H), based on their host specificity. While assemblages C–H are restricted to animals, A and B are zoonotic and infect both humans and other mammals (Cacciò et al., 2018; Zajaczkowski et al., 2021).

**Figure 1.**
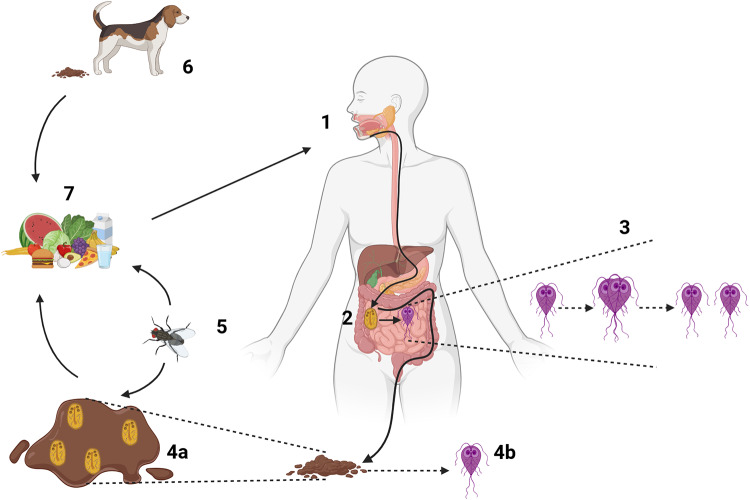
Life cycle of *G. lamblia*. Briefly: 1) Ingestion of food or water contaminated with mature cysts; 2) excystation and release of trophozoites in the small intestine; 3) multiplication of trophozoites by binary fission; 4a) excretion of cysts in feces; 4b) patients with diarrhoea may excrete trophozoites; 5) arthropods can transport cysts from feces to food; 6) feces from infected animals can contaminate food and water sources (zoonosis); 7) food and water contaminated with viable *Giardia* cysts capable of infecting humans. Figure 1 long description.

Transmission occurs via the faecal-oral route, either directly (e.g. person-to-person, zoonotic) or indirectly through the ingestion of contaminated water or food (Figure 1). The transmission of this intestinal protozoan through certain sexual practices has also been documented, with a higher prevalence observed in the homosexual population compared to heterosexual control groups (Schmerin et al., 1978; Escobedo et al., 2014). As with amoebiasis, *Giardia* has been identified as another intestinal infection linked to these practices within this population group (Ortega et al., 1984; Shelton, 2004). Infections in both humans and animals can be subclinical. When symptomatic, the infection can range from watery diarrhoea to a broad spectrum of enteric manifestations, notably flatulence, cramping and nausea. The condition is often characterized by malabsorption and greasy, foul-smelling stools, which can result in significant weight loss, fatigue and chills. Post-infectious complications may include growth and cognitive deficits (Allain and Buret, 2020; Rojas-López et al., 2022).

While precise global prevalence data for giardiasis are lacking, Painter et al. (2015) have reported an incidence rate of approximately 6.1 cases per 100 000 individuals in the general population, with notably higher rates observed in paediatric populations (Painter et al., 2015). Furthermore, available data highlight that the highest burden of clinical cases is consistently reported among children under 5 years of age (Cacciò and Sprong, 2011). However, the prevalence of human giardiasis is variable in developed and developing countries. In Latin America, this parasitosis is a public health problem due to various risk factors such as low socioeconomic conditions, overpopulated countries, with communities established in areas with difficult access to health services, the presence of hot climates, areas with hydrography of easy contamination, and inadequate management of human wastewater (Ibáñez-Cervantes et al., 2018; Fusaro et al., 2022; Sarria-Guzmán et al., 2022). Owing to these clinical and epidemiological factors, *Giardia* was integrated into the World Health Organization’s ‘Neglected Diseases Initiative’ (Savioli et al., 2006).

Within this framework, giardiasis presents 3 significant challenges to global health management. From a diagnostic perspective, the conventional method of microscopic stool examination, while the simplest, most economical and widely accessible, suffers from variable sensitivity, requires technical expertise and fails to discriminate between different *Giardia* assemblages. Conversely, immunological and molecular diagnostics offer greater speed and sensitivity but are associated with substantially higher costs compared to coproparasitoscopic techniques and require highly trained personnel for operation and interpretation (Soares and Tasca, 2016). In the therapeutic domain, clinical management relies on a limited pharmacological arsenal, primarily nitroimidazoles, which can induce adverse side effects and against which parasite resistance has been documented (Carter et al., 2018; Debnath et al., 2019). Finally, concerning prevention, no licensed vaccine for human use is currently available, severely limiting options for reducing transmission and the overall burden of infection (Davids et al., 2019).

These deficiencies underscore the urgent need to develop novel diagnostic, prophylactic and therapeutic tools grounded in a deeper molecular understanding of the parasite. This is precisely where the study of the *Giardia* proteome becomes pivotal. The study of the proteome enables the systematic identification of proteins expressed during distinct stages of the parasite’s life cycle. Furthermore, it reveals key molecular players in host-parasite interactions, including proteins directly involved in pathogenesis, virulence and immune evasion. This layer of functional information is instrumental for pinpointing potential candidates for therapeutic intervention or diagnostic biomarker development (Aziz et al., 2022).

Consequently, the review provides a functional perspective on the *G. lamblia* proteome, elucidating its critical role in infection mechanisms. It further offers a comprehensive update on the vaccine candidates, therapeutic targets and diagnostic tools derived from proteomic insights that are currently in development or hold promise for mitigating the impact of this parasitic disease.

### General characteristics of *G. lamblia*

**Figure 2.**
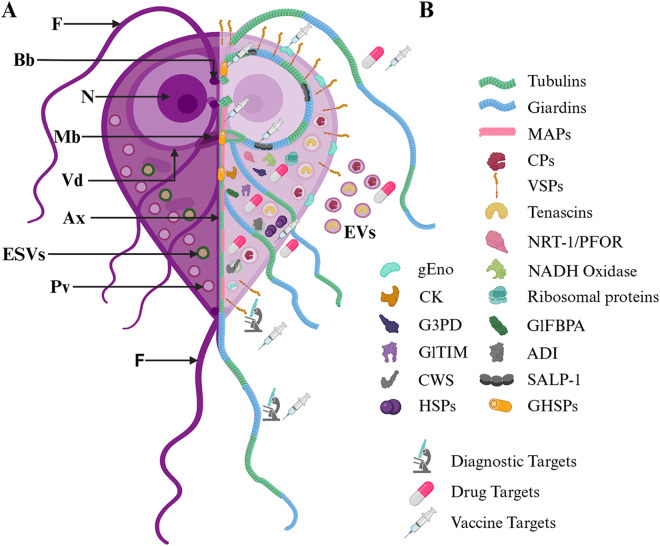
Morphology and key proteins of the *G. lamblia* trophozoite. (A) Structural schematic of a trophozoite depicting nuclei (N), flagella (F), basal bodies (Bb), median body (Mb), ventral disk (Vd), axoneme (Ax), encystation-specific vesicles (ESVs), peripheral vesicles (Pv) and extracellular vesicles (EVs). (B) Representation of major trophozoite proteins and their subcellular localization. The corresponding proteins are listed on the right. Proteins currently used or proposed as diagnostic, therapeutic and vaccine targets are highlighted; microscope, capsule and syringe, respectively. MAPs, microtubule-associated proteins, CPs, cysteine proteases, VSPs, variant-specific surface proteins, NRT-1, nitroreductase, PFOR, pyruvate-ferredoxin oxidoreductase, gEno, enolase, CK, carbamate kinase, GlFBPA, *G. lamblia* fructose-1,6-biphosphate aldolase, G3PD, glycerol-3-phosphate dehydrogenase, GlTIM, *G. lamblia* triose phosphate isomerase enzyme, ADI, arginine deiminase, CWS, cyst wall synthase, SALP-1, striated fibre assemblin-like protein, HSPs, heat shock proteins and GHSPs, *Giardia* head-stalk proteins. Figure 2 long description.

*G. lamblia* is a flagellated diplomonad protozoan (Scholey, 2008) characterized by its bilateral symmetry and the presence of 2 symmetrical nuclei at the trophozoite stage (Figure 2) (Kabnick and Peattie, 1990). While *Giardia* has an anaerobic metabolism (Schofield et al., 1990) and lacks classical mitochondria and a Golgi complex, it possesses mitochondria-like organelles known as mitosomes. These organelles, which lack DNA, are essential for the maturation of iron-sulphur proteins (Tovar et al., 2003; Martincová et al., 2015).

As mentioned above, *G. lamblia* is a multispecies complex with 8 recognized assemblages or genotypes in mammals, known as assemblages A–H (Monis et al., 2003). Assemblages A and B are the only genotypes known to infect humans, with mixed-assemblage infections appearing more frequently in developing countries (Almeida et al., 2010). Host specificity varies among domestic animals; livestock such as cattle, sheep and pigs are predominantly associated with assemblage E, whereas cats and dogs harbour a broad range, including assemblages A, B, C, D and F (Thompson and Monis, 2012; Cacciò et al., 2018).

### Morphology of *G. lamblia*

The *Giardia* life cycle comprises 2 primary stages – the trophozoite and the cyst – as well as intermediate encyzoite and excyzoite forms (Bernander et al., 2001; Reiner et al., 2008). The trophozoite is a pear-shaped, measuring 9–21 μm in length and 5–15 μm in width (Benchimol et al., 2023). Characterized by a broad anterior region and a tapered posterior end, the trophozoite features 8 flagella originating from basal bodies that emerge in posterior, ventral and caudal directions (Figure 2A) (Gadelha et al., 2020). The ventral side is concave and contains an adhesive disk (Figure 2A) surrounded by a lateral crest and a flange, which facilitates the attachment to the host’s small intestine epithelium (Hagen et al., 2011). Conversely, the convex dorsal surface contains 2 symmetrical nuclei positioned in the anterior half (Gadelha et al., 2020).

The trophozoite also contains a unique and characteristic structure known as the median body or midbody (Figure 2A), a microtubule-rich structure with a distinctive ‘crooked smile’ appearance (Dawson, 2010). While its definite function remains under research, it is hypothesized to serve as a tubulin reservoir, potentially regulating the parasite’s attachment and detachment kinetics (Piva and Benchimol, 2004). The cytoplasm also contains peripheral vesicles (PVs; Figure 2A), which constitute an endosomal-lysosomal system involved in nutrient uptake and exocytic activity. Additional essential components include the endoplasmic reticulum, ribosomes and glycogen granules. Notably, *Giardia* lacks conventional mitochondria, instead possessing mitosomes – organelles responsible for the assembly of iron-sulphur [Fe-S] clusters (Soltys et al., 1996; Tovar et al., 2003).

**Figure 3.**
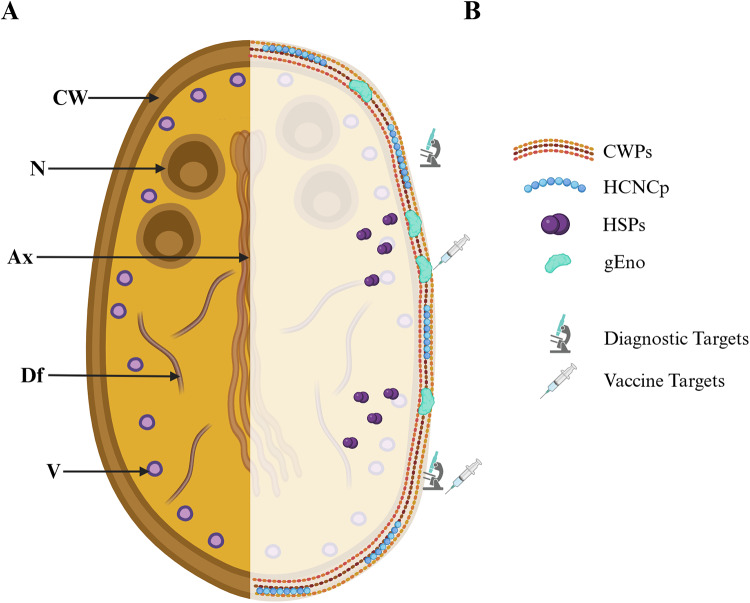
Morphology and key proteins of the *G. lamblia* cyst. (A) Structural schematic of a cyst depicting nuclei (N), cyst wall (CW), ventral disk fragments (Df), axoneme (Ax), vesicles (V). (B) Representation of major cyst proteins and their subcellular localization. Proteins currently used or proposed as therapeutic, vaccine and diagnostic targets are highlighted. CWPs, cyst wall proteins; HCNCp, high cysteine non-variant cyst protein; HSPs, heat shock proteins and gEno: enolase. Figure 3 long description.

In contrast, the cyst is the nonmotile, infective stage, optimized for environmental persistence and faecal-oral transmission (Adam, 2021). Cysts are oval-shaped (8–12 µm by 7–10 µm) and typically harbour 2–4 nuclei (Figure 3A). They are protected by a robust multi-layered cyst wall consisting of an outer filamentous glycocoat layer and an inner membranous layer (Figure 3A). This structure provides essential protection against osmotic stress and chemical disinfectants (Chávez‐Munguía et al., 2004; Chatterjee et al., 2010).

### The life cycle of *G. lamblia*

The infection initiates upon the ingestion of a low infective dose, requiring as few as 10–25 infective cysts (Figure 1.1, 1.7) (Wolfe, 1992). Following gastric passage, the acidic environment together with subsequent exposure to bile salts, pancreatic enzymes and an alkaline pH in the duodenum triggers excystation (Adam, 2001; Castillo-Romero et al., 2009). This process begins with the loosening of the filamentous cyst wall, followed by the enzymatic degradation of cyst wall proteins (CWPs) by giardial cysteine proteases (CPs) (Touz et al., 2002; Alvarado et al., 2022). Upon rupture of the cyst wall, the emerging excyzoite undergoes 2 successive rounds of cytokinesis without intervening DNA replication, ultimately yielding 4 binucleated trophozoites (Bernander et al., 2001; Jiráková et al., 2012). The resulting trophozoites colonize the proximal small intestine (Figure 1.2), where they utilize a ventral adhesive disk to anchor to the intestinal epithelium (Allain et al., 2017). Proliferation occurs via longitudinal binary fission (Figure 1.3), and recent evidence suggests that trophozoites form clusters within the small intestine and cecum, potentially to optimize nutrient acquisition or facilitate attachment (Barash et al., 2017).

Encystation is stimulated by trophozoites’ transit toward the lower ileum, often triggered by physiological cues (Fink et al., 2020). *In vitro*, this process is induced by cholesterol deprivation (Luján et al., 1996; Mi-ichi et al., 2022). Although the mechanism linking cholesterol sensing to differentiation is not fully understood, 1 hypothesis suggests that the inactivation or blockade of cholesterol receptors may be involved (Thomas et al., 2021).

Early-phase encystation is marked by the biogenesis of encystation-specific vesicles (ESVs), which transport CWPs to the plasma membrane for organized assembly of the cyst wall (Adam, 2001; Konrad et al., 2010). During this transformation, the cell (now termed an encyzoite) begins to round up, internalizing the ventral disk and flagella. The resulting mature cysts are excreted in the host’s feces and are immediately infective (Figures 1.4 a, b), capable of surviving in adverse conditions (Bernander et al., 2001; Midlej and Benchimol, 2009; Ankarklev et al., 2010). Transmission is primarily faecal-oral, and the zoonotic potential of *Giardia* is significant (Figure 1.6); companion animals serve as reservoirs, shedding cysts that are infectious to humans (Sprong et al., 2009). Furthermore, synanthropic arthropods (such as flies and cockroaches) play a critical role as mechanical vectors, facilitating the dispersal of mature cysts from waste to food sources (Graczyk et al., 2005), as shown in Figure 1.5 and 1.7.

## Proteins of importance during the trophozoite phase

### The structural proteome

The *Giardia* tubulin gene family is relatively simple, featuring only 2 α-tubulin and 3 β-tubulin genes (Kirk-Mason et al., 1989). This minimal genetic repertoire stands in sharp contrast to the expanded tubulin clusters found in other protozoa, such as *Leishmania* or *Trypanosoma brucei* (Landfear et al., 1983; Thomashow et al., 1983; Huang et al., 1984; Imboden et al., 1986). However, the parasite compensates for this simplicity through a sophisticated proteomic strategy, generating a vast array of functional isoforms via extensive post-translational modifications (PTMs) (Crossley and Holberton, 1983; Weber et al., 1997). A hallmark of this heterogeneity is the median body (Figure 2B), which serves as a dynamic microtubule reservoir (Dawson et al., 2007). By maintaining a localized balance of stable (acetylated and polyglycated) and dynamic (tyrosinated) tubulin, *Giardia* ensures a ready supply of subunits for the rapid assembly of the ventral disc and flagella (Figure 2B) during cytokinesis (Campanati et al., 2002; Park et al., 2021). This structural diversity is further corroborated by differential antibody labelling patterns, where immunoreactivity across different tubulin sources reflects the functional specialization of these proteomic variants despite their underlying genetic conservation (Coutinho Lopes et al., 2001).

The ventral disk´s structural proteome is dominated by giardins (Figure 2B), a parasite-unique family of cytoskeletal proteins that belong to the annexin superfamily. These proteins are indispensable for the architectural integrity and mechanical function of the ventral disk, an organelle that facilitates the parasite’s high-affinity attachment to the host’s intestinal epithelium (Figure 2B) (Crossley and Holberton, 1983; Weiland et al., 2003). Given that this microtubule-based attachment is a prerequisite for survival and pathogenesis within the host gut, the proteins mediating it are high-priority targets for therapeutic disruption (Elmendorf et al., 2003). The giardin proteome is categorized into 4 distinct subfamilies – α-, β-, γ- and δ-giardins – each exhibiting distinct structural and functional roles (Kim and Park, 2019).

The α-giardin subfamily is the most diverse, comprising 21 variants ranging between 29 and 38 kDa. Characterized by their ability to bind calcium and phospholipids, these proteins are primary mediators of membrane-cytoskeleton interactions (Morgan and M.P, 1995; Morgan and Fernandez, 1997). Proteomic mapping reveals highly specific spatial compartmentalization: α − 1 giardin (also known as taglin) localizes to the plasma membrane and ventral disk, whereas α − 2 and α − 19 are sequestered within specific flagellar pairs (Figure 2B) (Lev et al., 1986; Weiland et al., 2003). In contrast, β- and δ-giardins function as homologs to striated fibre assemblins (SFA). These proteins integrate into the microribbons extending from the ventral disc microtubules, providing the mechanical rigidity and structural framework necessary for intestinal attachment (Lourenço et al., 2012). While less conserved, γ-giardin remains an essential component for both ventral disk architecture and cell division coordination (Kim and Park, 2019). Collectively, proteomic analyses identify giardins as the major structural components of the ventral disk, with expression profiles suggesting additional roles in cellular differentiation and hot–cell interaction (Palm et al., 2005). Given their lack of human homologs and indispensable role in colonization, the giardin proteome represents an optimal landscape for therapeutic and diagnostic intervention (Figure 2B) (Steele-Ogus et al., 2021). Disrupting the assembly of these proteins could impair disc integrity, effectively neutralizing the parasite’s ability to maintain its niche within the host (Dawson, 2010; Nosala et al., 2020).

### Axonemal architecture and accessory proteomes

*Giardia* trophozoites possess 4 pairs of flagella, each powered by an axoneme (Figure 2A) exhibiting the canonical eukaryotic ‘9 + 2’ ultrastructure: 9 peripheral doublet microtubules surrounding a central singlet pair. These axonemes initiate from basal bodies situated inter-nuclearly in the anterior region of the cell (Elmendorf et al., 2003). Beyond the axoneme, *Giardia* flagellar apparatus is defined by a unique accessory proteome that confers biomechanical specificity. This includes paraflagellar dense rods, the marginal plates of the anterior flagella, the funis, a microtubular sheet associated with the caudal axonemes, and the basal bodies, which function as microtubule-organizing centres (Elmendorf et al., 2003; Hagen et al., 2011). These high-abundance structural proteins are essential for the complex, diverse beating patterns and biomechanical functions.

Tubulin is the primary structural protein (Figure 2B), but its assembly is strictly regulated. While α- and β-tubulin isoforms polymerize to form the microtubules, γ-tubulin is localized to the basal bodies, where it facilitates microtubule nucleation and axoneme initiation. Furthermore, specialized microtubule-associated proteins (MAPs), including centrin and tektin-like proteins, ensure the stability and dynamic regulation of these structures, allowing the flagella to withstand the physical stresses of the intestinal environment (Nohýnková et al., 2006; Sulimenko et al., 2022).

The biogenesis and maintenance of the flagellar apparatus depend on the intraflagellar transport (IFT) system. This machinery mediates the anterograde transport of protein cargo along the axoneme via kinesin-2 motor proteins, specifically GiKIN2a and GiKIN2b orthologs. Core IFT complex components, including homologs of IFT81 and IFT140, are localized to both flagella and basal bodies (Hoeng et al., 2008). These motors and transporters are critical vulnerabilities; disrupting IFT effectively halts flagellar assembly, rendering the parasite non-motile.

Protein-protein interactions and structural stability are further mediated by ankyrin repeat domain-containing proteins and coiled-coil proteins. GASP-180, localized to the intracellular portion of the anterior flagella, is implicated in structural support and flagellar regulation (Elmendorf et al., 2003). RIB72 – a highly conserved coiled-coil protein that stabilizes protofilament ribbons and dictates the mechanical properties of the axoneme (Hagen et al., 2011).

The giardial homolog of sperm-associated antigen 6 is localized to basal bodies and axonemes. Given its essential role in flagellar motility in higher eukaryotes, its presence in *Giardia* suggests a conserved proteomic mechanism for coordinating complex flagellar beating (Hagen et al., 2011).

## Pathoproteome of *Giardia*

Unlike many enteric pathogens, the virulence of *Giardia* is not mediated by the secretion of classic toxins. Instead, its pathogenicity arises from a sophisticated pathoproteome activated during complex parasite-host interactions at the intestinal mucosa. These virulence factors enable trophozoites to adhere, proliferate and orchestrate immune evasion (Nosala and Dawson, 2015). Central to *Giardia* virulence is its secretome, which serves as the primary interface for host manipulation. Through the release of excretion/secretion molecules delivered by ESVs, microvesicles (MVs) and exosomes, *G. lamblia* exerts cytotoxic and/or cytopathic effects on the host epithelium. This secretome is a complex mixture of catalytic proteins, metabolic enzymes and soluble mediators that target host cell receptors and extracellular matrix components. These secreted and surface proteins are categorized into 6 functionally distinct proteomic groups: toxin-like molecules, energy metabolism enzymes, cysteine-rich surface proteins, cathepsin B-like CPs, tenascins and cystatins (Evans-Osses et al., 2017; Ma’ayeh et al., 2017; Dubourg et al., 2018).

### The adhesive proteome and mechanical pathogenesis

The pathogenesis of *G. lamblia* begins with the high-affinity adhesion to and colonization of the intestinal epithelium, a complex process orchestrated by a specialized adhesive proteome and complex mechanical structures (Di Genova and Tonelli, 2016). The physical interface between the trophozoite and host enterocytes, maintained by the concerted action of the ventral disk and flagellar apparatus, induces mechanical damage to the host epithelium, generating apoptosis, disrupting microvilli and significantly reducing the total absorptive surface area (Troeger et al., 2007).

This damage impairs the intestinal uptake of essential nutrients, lipids, vitamins and carbohydrates, leading to clinical manifestations such as diarrhoea, steatorrhea and contributing to significant weight loss in chronic cases (Klimczak et al., 2024). Furthermore, Dawson and House (2010) highlighted the fundamental role of the *G. lamblia* cytoskeleton in driving this pathogenesis (Dawson and House, 2010). This cytoskeletal framework incorporates various proteins – including giardins, tubulins, actins, lectins and variant-specific surface proteins (VSPs) – which collectively function as molecular bridges that anchor the parasite to host intestinal epithelial cells (Figure 2B).

### CPs and cell damage

Genomic analyses of *G. lamblia* have identified a repertoire of at least 25 genes encoding CPs (Figure 2B), which serve as primary executors of the parasite’s proteolytic activity (Touz et al., 2002). Within this family, 21 genes encode Cathepsin-like proteases: 9 are categorized as Cathepsin B and 8 as Cathepsin L – both exhibiting endopeptidase and dipeptidyl peptidase activity – while a single Cathepsin C gene has been identified as a critical regulator of trophozoite encystation (Argüello-García et al., 2023). Due to their roles, Cathepsin B-type proteases represent a highly promising therapeutic target (Figure 2B) (Siqueira-Neto et al., 2018). Their potential is supported by the evidence that CP inhibition significantly reduces disease severity in other protozoan infections, such as those caused by *Trypanosoma* spp. and *Entamoeba* spp. (McKerrow et al., 2008). Furthermore, in acute giardiasis, Cathepsin B has been implicated in the development of chronic post-infectious sequelae, including irritable bowel syndrome (Allain et al., 2019).

Experimental models of trophozoite-cell interaction demonstrate that secreted CPs account for nearly the entirety of *Giardia-*derived proteolytic activity (Ma’ayeh et al., 2017). The predominant secreted protease, Cathepsin B (specifically Giardipain-1), orchestrates several pathogenic processes: immune evasion, direct cytotoxic activity and extensive epithelial damage via the disruption of intercellular junctions (Ma’ayeh et al., 2017). These proteases degrade essential components of the intestinal mucosa, such as Mucin-2, thereby facilitating trophozoite adhesion to the microvilli (Argüello-García et al., 2023). Furthermore, CPs induce structural alterations in villin – a key cytoskeletal protein of the microvilli – directly compromising epithelial integrity (Bhargava et al., 2015). Other critical junctional proteins targeted by these proteases include ZO-1, claudins, β-catenin and E-cadherin; the cumulative degradation of these proteins ultimately triggers enterocyte apoptosis (Liu et al., 2018).

Bioinformatic analyses have further revealed that the proteolytic scope of *Giardia* CPs extends to soluble mediators of the host innate immune response. This includes the degradation of secretory IgA (sIgA) produced by plasma B cells, as well as defensins and the neutrophil chemoattractant IL-8 produced by epithelial cells (Liu et al., 2019). By neutralizing these key immunological barriers, CPs facilitate persistent colonization and impede host-mediated clearance.

### VSPs and antigenic variation

To survive within the host and circumvent the immune response, *G. lamblia* employs a sophisticated mechanism of antigenic variation. This process is driven by the stochastic switching of highly immunogenic surface antigens termed VSPs (Figure 2B). VSPs are characterized as cysteine-rich, type-I integral membrane proteins. Their primary structural hallmark is the CXXC motif, which provides significant resistance to proteolytic degradation and facilitates the coordination of metal ions such as zinc and iron (Rodríguez-Walker et al., 2022). Each VSP molecule includes a conserved signal peptide, a single transmembrane domain and a highly conserved C-terminal cytoplasmic tail consisting of only 5 amino acids (CRGKA) (Adam et al., 1988). Collectively, VSPs form a dense, nearly impenetrable proteomic shield that covers the entire parasite surface (Figure 2B), including the ventral disk and flagellar apparatus (Pimenta et al., 1991).

The primary function of VSPs is to facilitate immune evasion through a mechanism of mutually exclusive expression. This process is regulated by the RNA interference (RNAi) pathway, which ensures the stabilization and translation of only 1 *vsp* mRNA transcript at any given time while targeting all other transcripts for degradation (Prucca et al., 2008). By periodically switching the expressed VSP during an infection, *Giardia* effectively ‘shifts’ its antigenic profile, allowing it to evade detection and clearance by host antibodies generated against the preceding VSP variant (Singer et al., 2019).

Comparative proteomic analyses indicate that virulent *G. lamblia* strains possess both a larger and more diverse VSP repertoire compared to less virulent strains, suggesting a direct correlation between VSP diversity and the parasite’s capacity for host adaptation and colonization (Emery et al., 2014). A notable example is VSP9B10A, a VSP that, when expressed in trophozoites, exhibits proteolytic activity resulting in a cytotoxic effect (Cabrera-Licona et al., 2017).

Beyond antibody evasion, recent evidence suggests that specific variants, such as VSP7, actively modulate the host cellular immune response. Specifically, VSP7 has been shown to reduce inflammatory cell death (pyroptosis) in macrophages, thereby suppressing host-mediated parasite-promoting chronic infection (Sun et al., 2023). However, VSPs represent a double-edged sword; while they mediate pathogenic effects, it has also been demonstrated that monoclonal antibodies (mAbs) targeting these proteins exhibit a cytotoxic effect against the trophozoite (Rivero et al., 2010). This dual nature positions VSPs as promising therapeutic and vaccine targets.

### Tenascins and immune evasion

Recent investigations have identified secreted tenascins (Figure 2B) as significant contributors to *G. lamblia’s* capacity for immune suppression and environmental persistence. These proteins are characterized by the presence of epidermal growth factor (EGF)-like domains, which allow the parasite to employ molecular mimicry to manipulate host signalling. Once secreted by trophozoites, tenascins interact with EGF receptors on intestinal epithelial cells, disrupting homeostatic signalling and impairing host immune surveillance (Dubourg et al., 2018).

This interaction exacerbates epithelial degradation by interfering with cell-cell adhesion and promoting the apoptosis of detached enterocytes, thereby compromising the integrity of the intestinal barrier. The secretion of tenascins (Figure 2B), coordinated with cathepsin B proteases and extracellular nucleases, constitutes a sophisticated proteomic strategy by which *Giardia* modulates the host environment to facilitate chronic colonization (Amat et al., 2017; Dubourg et al., 2018).

### Extracellular vesicles: vehicles for bioactive protein transport

Extracellular vesicles (EVs) are essential to this proteomic review due to their role as specialized delivery vehicles for pathogenic cargo (Figure 2B). Comprising both small extracellular vesicles (sEVs, < 100 nm; previously named exosomes) and MVs (up to 1 μm), these EVs facilitate complex communication between parasites and the host environment (Ma’ayeh et al., 2017; Pizarro et al., 2025). Their bioactive cargo includes a diverse repertoire of proteins, lipids and RNAs that collectively enhance parasite survival, adaptation and virulence (Faria et al., 2023).

Proteomic profiling has revealed that *Giardia-*derived EVs are highly enriched in cytoskeletal proteins and stress response molecules, underscoring their function in orchestrating immune evasion strategies (Gavinho et al., 2020). Evidence demonstrates that host macrophages internalize these vesicles, subsequently triggering the activation of Toll-like receptor 2 and the NLRP3 inflammasome. This interaction leads to the polarization of pro-inflammatory cytokines, specifically IL-1β and TNF-α (Zhao et al., 2021).

Moreover, a study by Yang et al. (2024) demonstrated that proteins transported by EVs modulated the gene regulation of Caco-2 cells upon exposure. These findings suggest that EVs play a pivotal role in host-parasite interactions, potentially amplifying pathogenic effects (Yang et al., 2024). Additionally, sEVs have been implicated in drug resistance, specifically to metronidazole. It has been shown that sEVs derived from resistant strains can enhance the genetic characteristics of wild-type parasites, effectively conferring drug resistance phenotypes (Pizarro et al., 2025).

As part of the secretome, enolase (gEno) has also been identified. This enzyme is expressed in the cytoplasm of trophozoites and has been localized on the surface of both trophozoites and cysts (Figures 2B, 3B). gEno acts as a virulence factor in host-parasite interactions by activating plasminogen and promoting necroptosis. This process is mediated by TNF-α and the apoptosis-inducing factor, ultimately leading to intestinal epithelial damage (Aguayo-Ortiz et al., 2017; Barroeta-Echegaray et al., 2022).

Furthermore, the lipidome of these vesicles – dominated by phosphatidylcholine, sphingomyelin and ceramides – suggests a sophisticated machinery for membrane trafficking and environmental responsiveness (Faria et al., 2023). Notably, EV biogenesis appears regulated by physiological cues such as luminal pH and calcium concentrations, indicating that *Giardia* uses EVs as dynamic sensors to respond to host-derived signals (Moyano et al., 2019).

The pathogenic repertoire of *G. lamblia* represents a complex network of molecular and cellular interactions that enable colonization, facilitate immune evasion and drive clinical disease. The protein families and vesicular systems described constitute the ‘molecular blueprint’ of giardiasis. Many of these candidates – particularly those involved in attachment, proteolysis and vesicular transport – represent high-priority targets for the development of next-generation vaccines and novel therapeutic interventions (Tables 2, 3).

### Proteomic induction of encystation

Encystation is a highly coordinated proteomic and metabolic transformation, culminating in the assembly of a protective cell wall. It involves transforming trophozoites into cysts, which is crucial for the parasite’s ability to resist environmental stress and infect new hosts. Morphologically, the *Giardia* cyst wall consists of 2 membranes (Figure 3A). The outer cyst wall is 0·3 to 0·5 μm thick and is composed of filaments that range between 7 and 20 nm in diameter (Luján et al., 1997). Regarding its composition, filaments are composed of approximately 37% of proteins, and the other 63% corresponds to (β1-3)-linked GalNAc homopolymer (Jarroll et al., 2001; Argüello-Garciá et al., 2009).

Encystation begins when the parasite continues its journey further down the small intestine, dividing it into 3 phases: (i) the stimulus for encystation and the regulation of encystation-specific gene expression, (ii) the synthesis of cyst wall components and (iii) the assembly of the extracellular cell wall (Luján et al., 1997). *In vitro*, this process takes place in 20–24 hours, after which the cyst is mature (Stefanic et al., 2006). During the first hours, encystation is induced by environmental cues, mainly related to cholesterol depletion, bile and alkaline environment (pH: 7.6–7.8) (Luján et al., 1997; Jarroll et al., 2001). Depletion of cholesterol causes an intracellular cAMP elevation, triggering the induction of encystation-specific genes such as the transcription factors GlMyb2, ARID/bright-like protein (Wang et al., 2007), Pax proteins (Wang et al., 2010), WRKY protein (Pan et al., 2009) and E2F (Su et al., 2011), responsible for the expression of CWPs (Luján et al., 1996; Shih et al., 2023).

Similarly, a proteomic analysis conducted by Kim et al. (2009) identified that *G. lamblia* expresses various heat shock proteins (HSPs) with distinct expression profiles depending on the parasite’s life stage (Figures 2B, 3B). Specifically, it was observed that Hsp70 and Hsp90 exhibit significantly higher expression levels during the encystation process (Kim et al., 2009). A subsequent study published by Nageshan et al. (2014), using transcriptomic and proteomic analysis, demonstrated that Hsp90 plays a pivotal role in the transition from the trophozoite to the cyst stage. This research revealed a 50% reduction in *hsp90* transcript levels in trophozoites compared to those observed during encystation (Nageshan et al., 2014). Nevertheless, further data are required to determine whether this upregulation in expression levels is intrinsically linked to the encystation process itself or represents a cellular stress response to harsh environmental stimuli, such as high bile concentrations (Kim et al., 2009; Steuart, 2010).

### Lectins and their protective action

Three structural CWPs (Figure 3B) have been identified (CWP1-3), which are synthesized in the endoplasmic reticulum (ER) and subsequently transferred to organelles termed ESVs (Figure 2A), and develop until almost all reach a uniform size, mature and are secretion competent (Štefanić et al., 2009; Midlej et al., 2013; Thomas et al., 2021). CWPs are acidic, cysteine-rich and characterized by 5 leucine-rich repeats (LRR)-like tandem repeats, targeted to the secretory pathway by amino-terminal signal peptides, possibly recognized by a cognate giardial receptor (Luján et al., 1997; Argüello-Garciá et al., 2009). While most CWPs have a molecular weight of 26 kDa, CWP2 is synthesized as a 39-kDa precursor that must be processed into a 26-kDa fragment. There are 27 gene sequences for the *Giardia* clan CA CPs family, with GlCP2 emerging as the most highly expressed. Previously, a cathepsin C-like enzyme known as encystation-specific cysteine protease (ESCP) was thought to be primarily responsible for this essential proteolytic processing. However, the prominence of GlCP2 was highlighted in this role. ESVs fuse with PVs to lower the pH, which reduces GlCP2 activity against CWP2 while potentially favouring ESCP. Furthermore, the ability of GlCP2 to fully degrade CWP2 under specific conditions suggests a secondary role for the enzyme in excystation once it is released into the extracellular space (DuBois et al., 2008; Chiu et al., 2010).

Initially, immature ESV membranes recruit peripheral matrix proteins, including b’COP, the Ypt1p-interacting protein homolog, Rab11 and dynamin-like protein (DLP). As encystation progresses, others such as the clathrin heavy chain (CLH) are recruited, with CLH and DLP redistributing specifically to the ESV (Marti and Hehl, 2003; Argüello-Garciá et al., 2009; Castillo-Romero et al., 2010). To maintain the integrity of the cyst wall, *Giardia* utilizes a stringent quality control system where misfolded cargo is processed via the proteasome and Sec61 translocon to allow ESV-to-ER retrograde transport within COPI-coated vesicles. During this cycle, chaperones such as immunoglobulin heavy chain-binding protein (BiP) and protein disulfide isomerases (PDI 1-3) ensure correct folding and oligomerization of CWP complexes (Stefanic et al., 2006; DuBois et al., 2008). As the mature ESV nears the plasma membrane, the calcium-binding protein (gGSP) maintains low intravesicular calcium levels to prevent premature assembly and regulate exocytosis (Lujan and Touz, 2003). Two proposed mechanisms facilitate the release of the cyst wall material: either the ESV fuses with PVs to acidify the cargo – limiting GlCP2 while potentially activating ESCP – or it undergoes fission into small secretory vesicles that fuse with the plasma membrane. Ultimately, the release of the antigenic [(glyco)polypeptide] matrix is achieved through an exocytosis process characterized by incomplete fusion, forming the protective barrier of the cyst (Hehl and Marti, 2004). Finally, Rho GTPases play a major role in coordinating the maturation and secretion of CWP to form the cyst wall (Krtková et al., 2016).

While vegetative trophozoites are typically covered by VSPs – cysteine-rich type I integral membrane proteins that protect against intestinal proteases – VSPs and CWPs exhibit distinct expression patterns and subcellular localization. VSPs switch during vegetative growth and are transported from the ER to the trophozoite plasmalemma, whereas CWPs are specifically targeted to the cyst wall. The High Cysteine Non-variant Cyst protein (HCNCp; Figure 3B), originally annotated as a large VSP, shares certain biochemical characteristics with VSPs, such as being an acidic, cysteine-rich, type I integral membrane protein. However, HCNCp is distinguished by numerous CxC motifs rarely found in VSPs and lacks the LRR motifs and higher molecular weight characteristic of CWPs (Davids et al., 2006). Interestingly, while HCNCp is highly expressed during encystation and localized in ESVs like CWPs, it eventually localizes to both the cyst wall and the cell body, unlike the exclusive wall localization of CWPs. This protein belongs to a larger genomic group of 61 high cysteine membrane proteins, which lack the VSP-specific C-terminal CRGKA motif and include VSP-like, EGF-like and transmembrane kinase-like molecules (Davids et al., 2006; Chiu et al., 2010).

Historical biochemical characterization has provided deep insight into the carbohydrate and protein matrix of the cyst. While early reports by Ward et al. (1985) suggested the presence of chitin (β-1,4-linked GlcNAc) based on wheat germ agglutinin binding and chitinase digestion (Ward et al., 1985), pioneering studies by Karr and Jarroll (2004) proved that the sugar homopolymer is composed of β-1,3-linked N-acetylgalactosamine (GalNAc). This metabolic shift is driven by an inducible enzyme activity, tentatively called cyst wall synthase (CWS), which exhibits very high affinity and specificity for UDP-GalNAc. Structural discoveries have revealed that in intact cyst walls, these unique GalNAc homopolymer fibrils are curled and form a complex lattice that is compressed into a narrow plane by bound proteins (Chatterjee et al., 2010). Notably, the LRR of CWP1 have been identified as a novel lectin domain specifically for these GalNAc fibrils, facilitating the structural assembly of the wall (Chatterjee et al., 2010). Furthermore, a cyst-specific glycohydrolase has been identified with the ability to degrade deproteinated GalNAc fibrils, suggesting a mechanism for wall disruption during excystation. While the homopolymer is synthesized from cytosolic UDP-GalNAc by a synthase, 5 enzymes in the soluble de novo pathway from glucose – including glucose-6-phosphate isomerase and glucosamine-6-phosphate deaminase – are up-regulated during encystation (Das and Gillin, 1996; Lopez et al., 2003; Faso et al., 2013). These homopolymers appear to be transported in specialized carbohydrate-positive vesicles (ECVs) (Chatterjee et al., 2010; Midlej et al., 2013).

Regarding the metabolism, the encystation process is highly energy-consuming. Transcriptome studies have revealed an increase in the expression of the glycolysis pathway for ATP generation during the early stages and a decrease to almost minimal expression during the late stages, when the cyst is already mature (Balan et al., 2021). It has been reported that phosphoglycerate kinase, pyruvate kinase, fructose-bisphosphate aldolase, glucokinase, glucose-6-phosphate isomerase and pyrophosphate-fructose 6-phosphate 1-phosphotransferase alpha subunit were more abundant at 4 hours post-encystation induction, while gEno decreases during this process (Faso et al., 2013). On the other hand, changes in lipid metabolism have also been observed, ranging from expression changes in homologues to cholesterol transporters in humans (Starkin lipid transporters), redistribution of lipid rafts and the formation of secretory vesicles associated with encystation. In turn, it has been observed that sphingolipid metabolic enzymes are transcribed only in cells undergoing encystation (Balan et al., 2021).

During the late phase, DNA replication co-occurs with nuclear division without cytokinesis, generating 2 nuclei precyst (4*2N), which replicates its DNA again, without cell division to form a 16N (4*4N) mature cyst (Einarsson et al., 2016). Previous studies demonstrated an up-regulation of meiotic-related genes during encystation, where DNA is suggested to recombine between homologous chromosomes using at least 3 meiotic-related genes (*Spo11, Hop1* and *Rad51*) in a process named diplomixis (Rojas-López et al., 2021). In addition, during this phase, up-regulation of methylation, acetylation and deacetylation enzymes, together with changes of histone methylation and acetylation, have been observed (Emery-Corbin et al., 2021). The histone methyltransferase 1 in *Giardia* induced an earlier and faster process with an upregulation of mRNA expression of CWPs during the early phase of the encystation process (Salusso et al., 2017). The study by Balan et al. (2021) showed that encystation is a highly regulated process, as observed in its results, but with no transcriptional controllers and few transcription factors. Therefore, various mechanisms have been identified for the regulation of this process, such as the identification of RNA-binding proteins, such as pumile-domain proteins, which are up-regulated during the encystation process. Although the function of these proteins in *Giardia* has not been completely elucidated, it has been proposed that, as in yeast, they could play a role in mRNA stability (Balan et al., 2021).

The entry mechanisms of lipids and other molecules in *Giardia* are carried out by receptor-mediated regulated endocytosis (EMR), in addition to other mechanisms such as pinocytosis or fluid-phase endocytosis (Rivero et al., 2013; Zumthor et al., 2016). When EMR is inhibited, the encystation process begins (Feliziani et al., 2022). The elucidation of the encystation pathway provides a robust framework for identifying novel therapeutic ‘choke points’, shifting the focus from broad-spectrum antiprotozoals to highly specific molecular targets. These strategies are primarily categorized into the inhibition of the cyst wall’s glycan matrix, the disruption of protein processing and the development of transmission-blocking vaccines.

## Protein targets for the diagnosis of giardiasis

The clinical diagnosis of giardiasis has evolved from classical morphology-based microscopy to highly sensitive immunoproteomic assays. Current methodologies include microscopic coproparasitoscopic analysis, immunological assays and molecular techniques designed to detect stage-specific antigens (Hooshyar et al., 2019). This review focuses exclusively on diagnostic platforms validated by international health institutions that rely on the detection of *G. lamblia* protein antigens.

The direct immunofluorescence assay (DFA) remains the gold standard for giardiasis diagnosis, serving as the reference method of emerging diagnostic platforms (Rishniw et al., 2010; National Center for Emerging and Zoonotic Infectious Diseases and Division of Parasitic Diseases and Malaria, 2024). The DFA involves processing faecal samples with a specific buffer, mounting them on slides and incubating them with fluorescent-labelled monoclonal or polyclonal antibodies to target high abundance (Riggs et al., 1983), primarily CWP1 and CWP2 (Figure 3B), which are the structural pillars of the cyst wall (Luján et al., 1995; Chatterjee et al., 2010). Commercial kits such as the Merifluor® (Aziz et al., 2001) and Para-Tect™ systems provide combined detection for *Giardia* and *Cryptosporidium*, demonstrating sensitivities and specificities ranging from 96 to 100% (Johnston et al., 2003; National Center for Emerging and Zoonotic Infectious Diseases and Division of Parasitic Diseases and Malaria, 2020).

The enzyme-linked immunosorbent assay (ELISA) or sandwich ELISA is high-throughput immunodiagnostic methods that detect soluble parasite proteins in faecal specimens. This methodology typically utilizes a microplate coated with capture antibodies; once the faecal antigen binds, a secondary mAb conjugated to horseradish peroxidase creates a colourimetric signal proportional to the parasitic load (Rosoff et al., 1989). For instance, assays like the *Giardia* CELISA kit (Cellabs, Brookvale) and the RIDASCREEN® *Giardia* assay employ mAbs to capture specific epitopes, often on CWPs (Boone et al., 1999; Beyhan and Taş Cengiz, 2017). In contrast, the Premier *Giardia lamblia* assay (Meridian Diagnostics, Inc.) utilizes a polyclonal antibody approach directed against the cyst wall proteome (Figure 3B), offering a broader detection spectrum by recognizing multiple epitopes (Fedorko et al., 2000). Other diagnostic kits, such as the ProSpecT™ *Giardia* microplate assay (Alexon, Inc.), were initially thought to detect a specific ∼65 kDa antigen termed *Giardia lamblia*-specific antigen 65 (GSA-65) (Rosoff and Stibbs, 1986b; Rosoff et al., 1989). However, subsequent research demonstrated that these assays target the CWP1 forming dimers with CWP2 (Luján et al., 1995; Boone et al., 1999).

Rosoff and Stibbs (1986a) first characterized GSA-65 in trophozoite and cyst extracts, as well as in faecal samples from infected patients. They identified the antigen as a glycosylated glycoprotein, a modification that confers proteolytic resistance, and suggested a critical structural role within the cyst wall (Rosoff and Stibbs, 1986a; 1986b). Later Boone et al. (1999) utilized the mAbs from the Alexon ProSpecT™ assay to identify CWP1, which has a molecular mass of 26 kDa (Boone et al., 1999). These findings aligned with the study by Luján et al. (1995), which employed specific antibodies for CWP1 (26 kDa) and CWP2 (39 kDa). Under non-reducing Western blot conditions, they observed that CWP1 and CWP2 form heterodimers, resulting in the characteristic 65 kDa bands (Luján et al., 1995).

In summary, ELISA-based assays are highly effective tools for the detection of CWPs. These diagnostics demonstrate robust clinical performance, with reported sensitivities ranging from 94 to 97% and specificities consistently between 99 and 100% (Maraha and Buiting, 2000; Johnston et al., 2003).

Immunochromatographic lateral-flow immunoassays, also known as rapid tests (RT), provide a decentralized diagnostic solution. The procedure involves mixing faecal samples with a specialized treatment buffer to solubilize the target antigens. Once applied to the test device, the sample migrates via capillary action across a nitrocellulose membrane. During this migration, the parasite proteins encounter anti-*Giardia* capture antibodies. If the target antigen is present, an immune complex forms and is subsequently immobilized by a secondary antibody at the ‘test line’, resulting in a visible coloured band (Chan et al., 2000).

These devices are primarily engineered to detect high-abundance structural CWPs such as CWP1 and CWP2 (Figure 3B), for example, ImmunoCard STAT!® and ColorPAC *Giardia*/*Cryptosporidium* (Chan et al., 2000; Garcia et al., 2003). Some RTs can identify antigens from various parasites, such as CerTest *Cryptosporidium/Giardia/Entamoeba* (Gutiérrez-Cisneros et al., 2011). This could give it advantages over other diagnoses.

Current reports indicate that these protein-based RTs achieve a sensitivity greater than 97% and a specificity of 100%, though performance may fluctuate in cases of low parasitic shedding (Katanik et al., 2001; Weitzel et al., 2007).

Beyond established markers, other structural and surface proteins show significant diagnostic potential but remain underutilized. Giardins, localized in the ventral disk (Figure 2B), are highly specific to *Giardia* (Weiland et al., 2003). VSPs such as VSP1267 and VSPH7 are exceptionally immunogenic; however, their high antigenic variability complicates their use as universal diagnostic markers (Figure 2B) (Garzon et al., 2021; Roshidi and Arifin, 2022). Moreover, other surface proteins, such as HCNCp, should be considered, following the rationale that most diagnostic assays focus on the detection of CWPs. While protein-based methods are highly effective, they require specialized equipment (fluorescence microscopes or microplate readers) and trained personnel. Furthermore, sensitivity may decrease during low-shedding phases of the infection, which may necessitate confirmatory testing (Katanik et al., 2001; Soares and Tasca, 2016). However, none of the currently available immunological tests can differentiate between *Giardia* assemblages, as they are designed to detect highly conserved proteins. A more detailed analysis could provide information on conserved and immunogenic epitopes that are unique to each assemblage. In this way, it would be possible to generate mAbs targeted against specific epitopes that enable differentiation between assemblages.

To address the limitations of protein-based assays, molecular methods targeting the *Giardia* genome, such as nested and real-time PCR, offer superior sensitivity. They are also able to differentiate between *Giardia* assemblages (Koehler et al., 2014). However, the cost of PCR remains high due to the need for a thermocycler and post-amplification processing, and the process needs to be carried out by specialized personnel (Soares and Tasca, 2016). As a cost-effective alternative for point-of-care testing, loop-mediated isothermal amplification (LAMP) has been proposed. LAMP provides diagnostic efficacy comparable to PCR but requires only a simple heating block, though it is currently limited to qualitative ‘yes/no’ detection, which can be a significant constraint for low-cost applications. Similarly, the CRISPR/Cas12a system has been extensively employed for pathogen detection. Recent studies have successfully utilized the CRISPR/Cas12a platform for the diagnosis of giardiasis, demonstrating sensitivity and specificity comparable to those of nested PCR (Zhao et al., 2024).

## Proteins as therapeutic targets

In recent years, research on *Giardia* has increased significantly, probably due to the re-emergence in industrialized countries or because of the World Health Organization listing giardiasis in the Neglected Disease Initiative since 2004 (Savioli et al., 2006). However, the number of drugs available against this infection has not changed substantially in recent decades (Escobedo et al., 2016). The primary pharmacological defence against giardiasis remains the 5-nitroimidazoles (metronidazole, tinidazole, ornidazole and secnidazole; Table 1).

**Table 1.** Antigiardial drugs: mechanisms of action against key cellular targets Table 1 long description.

Drug	Primary proteomic target	Main mechanism of action	References
Nitroimidazoles	PFOR/ferredoxin	Activation into radicals that damage DNA and proteins.	Gardner and Hill (2001); Müller et al. (2007); Vivancos et al. (2018); Mørch and Hanevik (2020)
Benzimidazoles	β-tubulin	Inhibition of cytoskeletal polymerization, preventing the formation of microtubules and affecting cell motility and structure.	Jiménez-Cardoso et al. (2009); Vivancos et al. (2018)
Nitazoxanide	NRT-1, PFOR, quinone reductase	Interference with energy metabolism.	Müller et al. (2007); Vivancos et al. (2018); Argüello-García et al. (2020); Mørch and Hanevik (2020)
Paromomycin	Ribosomal proteins	Inhibition of *Giardia* protein synthesis, leading to an erroneous reading of mRNA.	Argüello-García et al. (2020); Mørch and Hanevik (2020)
Furazolidone	NADH oxidase	Superoxide radical generation; inhibits encystation.	Brown et al. (1996); Gardner and Hill (2001)
Chloroquine	Ventral disc/adhesion proteins.	Disruption of intestinal attachment/colonization	Gardner and Hill (2001); Carter et al. (2018)

Metronidazole (MTZ) acts as a prodrug reduced via the parasite’s electron transport system, specifically involving pyruvate: ferredoxin oxidoreductase (PFOR) and ferredoxin (Table 1, Figure 2B). This reduction generates toxic free radicals that destabilize DNA and impair protein function, leading to trophozoite death (Gardner and Hill, 2001; Mørch and Hanevik, 2020). However, MTZ causes significant side effects and inhibits aldehyde dehydrogenase, necessitating the avoidance of alcohol to prevent disulfiram-like reactions (Vivancos et al., 2018). The rise of nitroimidazole-refractory giardiasis highlights a complex, heterogeneous picture of resistance. Treatment failures are common (up to 20%), clinical resistance is proven and *in vitro* resistance can be induced so that parasites grow in clinically relevant levels of MTZ (Gardner and Hill, 2001; Tejman-Yarden et al., 2011). Resistance is not tied to a single mutation but to the altered expression of oxidative and reductive enzymes (Leitsch et al., 2011; Nillius et al., 2011). The most consistent marker of resistance is the downregulation of nitroreductase-1 (GlNR1; Figure 2B), the major enzyme responsible for drug activation, whereas GlNR2 appears to play a detoxifying role (Müller et al., 2007; Nillius et al., 2011). Furthermore, the parasite alters its core metabolism by downregulating PFOR2 and shifting its thioredoxin reductase system (Leitsch et al., 2011; Leitsch, 2015). Even when activating proteins are present, a depletion of crucial cofactors like FAD and NADPH can prevent MTZ conversion, demonstrating how *Giardia* strategically ‘dims’ its redox proteome to achieve tolerance (Leitsch, 2015; Müller et al., 2015; Krakovka et al., 2022). Furthermore, evidence suggests that *Giardia* can modulate its proteome via sEVs shared among parasites, potentially disseminating resistance traits across the population (Pizarro et al., 2025).

Other compounds target different elements of the *Giardia* proteome. Benzimidazoles (albendazole and mebendazole) bind to β-tubulin (Table 1; Figure 2B), inhibiting cytoskeletal polymerization and interfering with glucose uptake (Jiménez-Cardoso et al., 2009; Vivancos et al., 2018). Nitazoxanide (Nitrothiazole) offers a multifactorial mechanism by inhibiting essential enzymes, including NRT-1, PFOR and quinone reductase (Table 1) (Argüello-García et al., 2020). Paromomycin, an aminoglycoside, is one of the few antiparasitics prescribed during pregnancy, due to its poor intestinal absorption, but its activity is lower than that of nitroimidazoles, which act by inhibiting *Giardia* protein synthesis (Table 1), though its efficacy is lower in refractory cases than quinacrine and furazolidone (Mørch and Hanevik, 2020). Furazolidone serves as a potent nitrofuran derivative that utilizes NADH oxidase for its reduction, producing superoxide radicals that damage DNA and cellular organelles (Table 1). This oxidative stress specifically impairs the trophozoites’ ability to differentiate into cysts (Brown et al., 1996; Gardner and Hill, 2001; Carter et al., 2018). Finally, chloroquine, originally introduced as an antimalarial in 1930, has demonstrated clinical efficacy. It’s hypothesized that the proteomic mechanism involves compromising the trophozoite’s physical ability to attach and colonize the intestinal epithelium, likely by disrupting proteins associated with the ventral disc (Table 1) (Gardner and Hill, 2001; Carter et al., 2018).

The search for new therapies has shifted toward targets that are structurally unique to *Giardia* or essential to its survival regardless of its redox state. Since metabolic plasticity allows the parasite to bypass traditional drugs by simply ‘dimming’ its redox pathways, researchers are now prioritizing the identification of parasite-specific biomolecular targets. The arginine dihydrolase pathway is a premier candidate because its first and final enzymes, arginine deiminase (ADI) and the carbamate kinase (CK), are absent in humans. Arginine deiminase catalyses the initial hydrolysis of L-arginine to L-citrulline and ammonia. Studies involving RNAi knock-down of ADI result in non-viable trophozoites (Table 2), and it may serve as a virulence factor by depleting host arginine to evade the immune response (Li et al., 2009). CK catalyses the conversion of carbamoyl phosphate and ADP into carbamate and ATP; its essentiality to parasite energy production and its absence in the host genome make it a highly selective target (Table 2; Figure 2B) (Chen et al., 2012; Galkin et al., 2014). Similarly, the parasite’s reliance on glycolysis highlights Class II fructose 1,6-bisphosphate aldolase (GlFBPA; Table 2; Figure 2B) (Galkin et al., 2007; Méndez et al., 2019). Because humans utilize Class I aldolases, the structural divergence of this protein allows for the design of specific inhibitors (Muller, 1988; Gefflaut et al., 1995). Even in conserved proteins like triose phosphate isomerase (GlTIM), the *Giardia* variant possesses a specific cysteine at position 222 (C222) that serves as a unique ‘Achilles’ heel’ for parasite-specific inactivation (Table 2; Figure 2B) (Enriquez-Flores et al., 2008; García-Torres et al., 2016).

**Table 2.** New pharmacological proposals against giardiasis Table 2 long description.

Therapeutic candidate	Molecular target	Pathway/mechanism	Rationale for selectivity	References
RNAi (experimental)	Arginine deiminase (ADI)	ATP generation/immune evasion	Knock-down leads to non-viable trophozoites.	Li et al. (2009)
CK modulators	Carbamate kinase (CK)	Arginine dihydrolase: Inhibits ATP production from L-arginine.	Protein is completely absent in the human genome; essential for parasite ATP. Target is completely absent in humans.	Chen et al. (2012); Galkin et al. (2014)
GlFBPA inhibitors	Class II fructose-1,6-bisphosphate aldolase	Glycolysis: Selective inhibition of the parasite’s Class II enzyme.	Structural divergence: *Giardia* uses Class II, while humans use Class I aldolases.	Muller (1988); Galkin et al. (2007); Méndez et al. (2019)
GITIM inhibitors (C222)	Triose phosphate isomerase (GlTIM)	Glycolysis: Targets a specific cysteine residue (C222) unique to *Giardia*.	Target contains a unique cysteine (C222) not found in the human homolog.	Enriquez-Flores et al. (2008); García-Torres et al. (2016)
HSPs inhibitors (prodrug SNX-5422)	Heat shock proteins 90 (Hsp90)	Transition from the trophozoite to the cyst stage	Alteration of the encysting process	Nageshan et al. (2014); Debnath et al. (2014); Palma et al. (2019)
Phosphonoxins	Cyst wall synthase (CWS)	Encystation: Inhibits the formation of the insoluble GalNAc fibrillar lattice.	Targets glycosyl transferase activity; dual-action (trophozoite/cyst) inhibition.	Suk et al. (2007)
NBDHEX	Glycerol-3-phosphate dehydrogenase (G3PD)	Redox/metabolism: Inhibits redox balance and potentially thioredoxin reductase.	High affinity binding; inhibits multiple targets, including Thioredoxin reductase. Higher *in vitro* efficacy than MTZ.	Lalle et al. (2015); Camerini et al. (2017)

The process of encystation provides a final framework for proteomic intervention. Hsp90 plays a pivotal role in the developmental transition from the trophozoite to the cyst stage in *G. lamblia* (Nageshan et al., 2014). Consequently, the pharmacological inhibition of Hsp90 (Table 2; Figures 2B, 3B) has garnered significant attention, as this chaperone is essential for the viability of numerous protozoan parasites (Palma et al., 2019). In 2014, Debnath and colleagues evaluated and compared the antigiardial activity of the prodrug SNX-5422 – a benzamide derivative – against benzonitrile intermediates involved in benzamide synthesis. Unlike the nitrile derivatives, the prodrug demonstrated robust efficacy against giardiasis in a murine model. Mechanistically, the benzamide moiety establishes critical hydrogen bonding interactions within the ATP-binding pocket of Hsp90, a feature absent in the benzonitrilic analogues (Table 2). These findings suggest that the potent activity of SNX-5422 against *Giardia* is primarily mediated by the targeted inhibition of Hsp90 (Debnath et al., 2014).

The cyst wall is a composite of CWPs and a β-linked GalNAc homopolymer synthesized by CWS (Karr and Jarroll, 2004). While phosphonoxins were designed as transition-state inhibitors of CWS (Figure 3B), proteomic and functional studies of lead compound 10 f show it inhibits both vegetative trophozoite growth and encystation (Suk et al., 2007). This suggests that phosphonoxins may target broader glycosyl transferase pathways essential to both life stages (Table 2).

Another promising candidate is glycerol-3-phosphate dehydrogenase (gG3PD), a key enzyme in redox and fatty acid metabolism (Table 2). The compound NBDHEX (6-(7-nitro-2,1,3-benzoxadiazole-4-ylthio) hexanol), a new class of antitumor components, binds to gG3PD and potentially thioredoxin reductase (Table 2; Figure 3B), offering a multi-objective approach that is more effective *in vitro* than metronidazole (Lalle et al., 2015; Camerini et al., 2017).

The transition from broad-spectrum antiprotozoals to highly specific molecular inhibitors represents a paradigm shift in the management of *G. lamblia*. As traditional therapies like metronidazole face increasing challenges from complex, multi-enzymatic resistance mechanisms, the proteins identified within the arginine dihydrolase pathway, glycolytic machinery and the encystation programme offer a more resilient framework for drug development. By targeting enzymes like CK or CWS, which have no human homologs, host toxicity can be minimized while bypassing the parasite’s ability to ‘dim’ its redox activation pathways. Moving forward, the clinical community must prioritize the identification of reliable molecular markers for drug resistance to replace current empirical treatment strategies. Ultimately, a multi-targeted approach – combining novel inhibitors with prophylactic vaccines – holds the potential to transform giardiasis from a persistent global health threat into a manageable and, eventually, eradicable disease.

## Giardiasis vaccines: their target antigens, new vaccine platforms, adjuvants and immunostimulants

As one of the most prevalent global protozoan infections, *G. lamblia* presents a significant public health challenge, exacerbated by rising resistance to frontline therapies like metronidazole and albendazole (Argüello-García et al., 2020; Pech-Santiago et al., 2022). In addition to drug resistance, other factors can also contribute to treatment failure against *Giardia*, such as reinfection, insufficient or inappropriate drug administration and immunosuppression (Leitsch, 2015). Immunocompromised individuals are more vulnerable to severe and chronic infections, which also makes them chronic carriers of giardiasis (Wang and Greene, 2025). Consequently, vaccines represent a highly viable strategy for controlling outbreaks, reducing antiparasitic drug use and mitigating clinical complications, such as the severe weight loss associated with protozoan-induced intestinal damage.

For giardiasis immunization, various antigens have been explored, ranging from inactivated trophozoites, whole extracts, or specific antigens such as the GLSA-56 (surface-associated antigen, 56 kDa), VSPs, CWP-2, α1 and β-giardin (Table 3; Figures 2B, 3B), and a uridine phosphorylase-like protein. For most of these antigens, specific immunoglobulins have been detected in the sera of immunized mice, gerbils, dogs, or cats. In addition, a reduction in cyst shedding or trophozoite load to subunit vaccines targeting specific proteomic components.

**Table 3.** Proposed vaccines against giardiasis Table 3 long description.

Species or strain	Antigen	Formulation	Route of administration	Evaluation of the immunity	Efficacy and safety	Reference
*G. muris*	Trophozoites	Trophozoites + freund complete adjuvant.	Injection into the peritoneal cavity and hind footpads.	Challenged assay by cysts in oesophagus of mice and measurement of cyst excretion in feces.	80% of immunized mice do not pass faecal cysts for 8 weeks.	Roberts-Thomson and Mitchell (1979)
*G. lamblia*	Trophozoites	Trophozoites + PBS + 10% of TYI-S-33 medium.	Injection into the stomach.	Increased serum IgG and IgM of immunized mice	Not evaluated.	Gottstein et al. (1990)
*G. lamblia*	Protein GLSA-56 (or 65 kDa antigen)	Multilamellar phosphatidylcholine liposomes + GLSA-56 protein	Subcutaneous injection and 7 days after intraesophageally by catheter.	Increase of IgA and IgG anti-GLSA-56 in serum. Increase in CD8^+^ and CD4^+^ lymphocytes.	The trophozoite load was low and was eliminated more rapidly in vaccinated animals after challenge.	Vinayak et al. (1992)
				Challenged assay by cysts in oesophagus of mice and measurement of trophozoite load in the jejunum.		
*G. lamblia*	Proteins of axenically cultured trophozoites *G. duodenalis* (sheep isolate)	Proteins + Alum adjuvant	Subcutaneous injection in the dorsal thoracic area.	Increased specific serum and mucosal IgG and IgA, respectively.	Clinical signs were not seen or were significantly reduced. Adverse reactions were not observed. Vaccination reduced the proportion of animals shedding cysts (30% for vaccinated kittens and 5% for vaccinated puppies). Also, reduced trophozoites in vaccinated animals.	Olson et al. (1996), Olson et al. (1997)
				Challenged assay by trophozoite load in duodenum by surgery.		
*G. lamblia*	A peptide fragment of VSP H7	Peptide of VSP H7 + attenuated *S. typhimurium* (gal Emutant) strain LT2M1C.	Intragastric inoculation.	Increased serum IgG and IgM of immunized mice.	Not evaluated.	Stäger et al. (1997)
*G. lamblia, G. muris*	Soluble antigenic extracts of *G. lamblia* and *G. muris* trophozoites	Only extracts in PBS.	Intraperitoneal inoculation.	Increased IFN-γ, IL-4 and IL-5.	Not evaluated.	Djamiatun and Faubert (1998)
*G. lamblia*	Trophozoites killed (GiardiaVax®)	Trophozoites killed + adjuvant	Approved vaccine by subcutaneous injection	Challenged assay by *G. muris* cyst loaded in digestive system.	Clinical signs resolved between 16 and 42 days post-vaccination and cessation of faecal cyst shedding was between 21 and 70 days. Adverse reactions to the vaccine were not observed in dogs. Trophozoites could not be found in small intestine	Olson et al. (2000, 2001)
*G. intestinalis* WB strain ATCC 30957	CWP2 protein	CWP2 + CT adjuvant	Oral immunization	Increased serum and IgG1 and IgG2a. Immunization showed high levels of IL-2, IL-10 and TGF-β in the spleen and PPC. Challenged assay by *G. muris* cyst loaded in digestive system.	Vaccinated mice shed fewer cysts.	Larocque et al. (2003)
*G. intestinalis* WB strain ATCC 30957	26 kDa fragment of CWP2 protein (M6-CWP2 fusion protein)	M6-CWP2 + *Streptococcus gordonii* PL16	Intragastric inoculation	Increased serum IgG and faecal IgA.	Vaccinated mice shed fewer cysts (around 70% less).	Lee and Faubert (2006)
				Challenged assay with live *G. muris* cysts.		
*G. intestinalis* WB strain ATCC 30957	CWP2 protein	CWP2 + *Salmonella typhimurium* (STM1 strain)	Oral immunization	Increased the numbers of CD4^+^ T-cells and CD19^+^ B-lymphocytes in the PPC and MLN. Increased serum IgG2a and faecal IgA and IgG. Challenged assay by oral load live *G. muris* cysts.	Vaccinated mice shed fewer cysts (around 60% less).	Abdul-Wahid and Faubert (2007)
*G. intestinalis*	CWP2 recombinant protein	*L. lactis*-CWP2 or *S. gordonii*-CWP2	Oral immunization	Increased the numbers of CD4^+^ T-cells, CD19^+^ B-lymphocytes and IFN-gamma in the PPC and MLN. Increased IgG and IgA in the small intestine.	Vaccinated mice shed fewer cysts (70–90% less). No adverse signs were noted, and no animals died during challenge.	Lee et al. (2009)
*G. intestinalis* WB strain ATCC 50803	α1-giardin or ornithine carbamoyl transferase or α-enolase (this final protein does not confer protection)	One protein of *G. intestinalis* + attenuated *Salmonella enterica* Serovar *Typhimurium*	Oral immunization	Increased serum IgG and faecal IgA (α1-giardin and α-enolase).	Mice vaccinated with α1-giardin had fewer trophozoites in the small intestine.	Jenikova et al. (2011)
				Challenged assay with live *G. lamblia* GS/M trophozoites by oral load.		
*G. intestinalis* WB strain ATCC 50803	CWP2 peptide (aa 248–363) and α1-giardin.	Two proteins + attenuated *Salmonella typhimurium* SL7027	Oral immunization	Increased serum IgG2a and faecal IgA. Challenged assay with live *G. lamblia* C2 trophozoites by oral load.	Vaccinated mice shed fewer cysts (around 90% less) and reduced the trophozoite load in small intestine (80%).	Feng et al. (2016)
*G. intestinalis* WB strain ATCC 50803	Mixture of purified VSP	VSP + PBS	Oral immunization	Increased serum IgG and faecal IgA. Immunization showed high levels of IL-17, IL-6, IL-4 and IL-5, without obvious signs of inflammation (IFNγ, IL-1b, TNF) in the spleen, PPC and MLN. Challenged assay by orogastric inoculation of trophozoites in gerbils and measurement of trophozoite or cyst excretion in feces.	Only unvaccinated and infected mice showed evident signs of giardiasis.	Serradell et al. (2018)
*G. intestinalis* WB strain ATCC 50803	α1G: α1-giardin; α11G: α11-giardin; UPL-1: uridine phosphorylase-like protein; and protein 21.1	One protein of *G. intestinalis* + cholera toxin	Intranasal immunization	Increased serum IgG with the 4 proteins immunized; and increased IgA (only with α11 G and UPL-1) of small intestine.	Vaccinated mice with the 4 proteins reduced the trophozoite load in small intestine, and vaccinated gerbils only with α1 G.	Davids et al. (2019)
				Challenged assay with *G. lamblia* trophozoites (WB/C6 or GS/M) by oral gavage to mice or gerbils.		
*G. lamblia* WB strain	VSP 1267	VSP1267 + VLP (HA and NA of H5N1) + Alum	Oral immunization	VSP stimulates host innate immune responses in a TLR-4 dependent manner. Increased serum IgG and faecal IgA. Challenged assay with VSP + VLP by oral gavage to mice.	Not evaluated.	Serradell et al. (2019)
*G. intestinalis* strain ATCC 50581	Trophozoites GS-5G8 (+) strain (increased VSP 5G8 protein expression level)	Trophozoites + Freund’s adjuvant	Intraperitoneal inoculation	Increased serum IgG and faecal IgA. Serum antibody from infected and reinfected mice exhibited specific agglutination of trophozoites *in vitro*. GS-5G8 (+)-immunized mice showed higher CD19 + infiltrating cell levels.	Not evaluated.	Garzon et al. (2020)
*G. intestinalis* strain ATCC 50581	α1-giardin and β-giardin	Protein + cholera toxin	Intranasal immunization	Increased serum IgG and faecal IgA.	Vaccinated gnotobiotic mice reduced the trophozoite load in small intestine.	Ihara et al. (2024)
				Challenged assay with *G. lamblia* trophozoites by oral administration to gnotobiotic mice.		

PBS, phosphate-buffered saline; VSPs, variant-specific surface proteins; CWP, cyst wall proteins; PPC, Peyer’s patch cells; MLN, mesenteric lymph nodes; VLP, virus-like particles.

Surface proteins represent a promising source of antigens, as key host effectors, such as intestinal secretory IgA, can bind directly to these antigens within the intestinal lumen and interfere with the epithelial adhesion of trophozoites. Some surface proteins of the *Giardia* trophozoite include giardins, VSPs, (SFA)-like protein (e.g. SALP-1) and *Giardia* head-stalk proteins (GHSPs; e.g. GASP-180) (Figure 2B). However, many of these surface proteins have not been tested as vaccine antigens in animal models; in fact, research has been largely limited to protein characterization (Palm et al., 2003; Bae et al., 2009; Rivero et al., 2010). In some cases, antibodies against these proteins have been identified in animals or humans infected with *Giardia* trophozoites (Lopez-Romero et al., 2017; Hjøllo et al., 2018). A similar situation exists for CWPs; CWP1 and CWP3 have been well characterized, but they have not yet been evaluated as vaccine antigens (Luján et al., 1995).

Novel antigenic candidates have been proposed, including lectins, enolase, giardins, tubulins and HSPs (Figures 2B, 3B), among others (Garzon et al., 2021). Despite these efforts, the only licensed vaccine is GiardiaVax – a chemically inactivated trophozoite preparation exclusively for veterinarian use (Table 3) (Anderson et al., 2004). No human vaccine is currently approved by the U.S. Food and Drug Administration or the European Medicines Agency. The complexity of the *Giardia* life cycle presents a major hurdle, as antigen expression differs considerably between the trophozoite and cyst stages (Lingdan et al., 2012). A vaccine containing at least 2 antigens – 1 for each stage – could provide dual protection against both disease and transmission (Feng et al., 2016). In this regard, Feng’s group demonstrated that a bivalent vaccine, proteins α1-giardin and CWP2, protected mice against colonization by the *Giardia* trophozoite and, at the same time, reduced cyst formation in the host (Feng et al., 2016). However, the CWP2 protein exhibits dozens of amino acid substitutions and deletions when derived from different assemblages, A and B, isolated from human samples (Radunovic et al., 2017). This suggests that several CWP2 antigens should be used to develop a *Giardia* vaccine that provides cross-protection against both assemblies. Additionally, because assemblages A and B infect dogs, cats and rats, a veterinary vaccine could significantly reduce zoonotic transmission (Cacciò and Ryan, 2008). Therefore, the development of both veterinary and human vaccines is equally important.

One of the first questions in vaccine development is what type of antigens will be tested to generate the vaccine, or whether the whole pathogen will be used. In this regard, there are more reports of *Giardia* vaccines using 1 or more antigens than reports using the whole parasite. This review lists the antigens already used (Table 3) and proposes new antigens for investigation (Figures 2B, 3B). However, an effective vaccine not only depends on a highly immunogenic antigen, but also requires a suitable platform that provides stability, durability and acts as an adjuvant. There is a growing opportunity to utilize next-generation vaccine platforms to enhance immunogenicity. Emerging strategies include cationic solid-lipid nanoparticles, Virus-Like Particles with surface proteins of different protozoan (Saljoughian et al., 2013; Serradell et al., 2019), DNA and mRNA-based vaccines, which have shown promise in other protozoan models like *Malaria* and *Leishmania* (Dumonteil, 2007; Wu et al., 2025), among others. These platforms, combined with novel antigens or compounds (polythene glycol, protamine, poly-lactic acid, MF59 adjuvant, among others), could provide the safety and efficacy profiles necessary for high-risk populations, specifically children under 5 years of age.

New antigens are proposed here, such as MAPs, CPs, nitroreductase-1 and pyruvate-ferredoxin oxidoreductase, among others, present in the trophozoite (Figure 2B). Cyst antigens such as high cysteine non-variant cyst protein or CWPs are also proposed (Figure 3B). Generally, subunit vaccines are known to be safe in nature, but they are mostly found to be incapable of generating the optimum immune response. Hence, there is a great possibility of improving the potential of a vaccine in formulation with novel adjuvants, which can effectively impart superior immunity. In this regard, chitosan is a mucosal adjuvant that serves both as an immunostimulant and as a vehicle for oral administration. Chitosan facilitates resistance to both acidic and basic pH levels (Zhu et al., 2021); therefore, chitosan would facilitate the development of an orally administered *Giardia* vaccine, as it would protect the antigen during its journey from the acidic environment of the stomach to the basic pH of the intestine, thus enhancing the antigen’s bioavailability in the intestinal mucosa, as occurs in a natural infection.

Bacterially derived adenosine diphosphate (ADP)-ribosylating enterotoxins are the most characterized mucosal adjuvants. This class of adjuvants comprises cholera toxin and *Escherichia coli* heat-labile enterotoxin. These toxins stimulate both a humoral and cellular immune response, producing antigen-specific IgA antibodies and long-lasting memory cells for vaccine antigens when administered via the mucosal route (Elson and Ealding, 1984; Clements and Norton, 2018). However, *E. coli* heat-labile enterotoxin has caused few cases of transient facial paralysis in humans when administered nasally, so its use is limited to animal studies (Mutsch et al., 2004). Nevertheless, there are safe adjuvants widely used in humans, such as MF-59 (Vesikari et al., 2012; Nolan et al., 2014), aluminium as mineral salt (Krauss et al., 2022), AS03, AS04, RC-529 (Verma et al., 2023) and CpG ODN (Otsuka et al., 2019). Any of these adjuvants can be used in the design of safe *Giardia* vaccines to enhance the immune response.

Therefore, an ideal *Giardia* vaccine would contain antigens from both the trophozoite and the cyst; allow cross-protection between assemblages; contain immunostimulants and adjuvants that increase their immunogenicity; include a biomaterial that promotes their bioavailability, stability and potency; and be safe and effective. Ideally, this vaccine should be modified and designed for both veterinary and human use.

## Conclusions and future directions

The functional analysis of the *G. lamblia* proteome presented in this review underscores the complexity of both the parasite’s biology and its pathogenic strategies within an apparently simple life cycle (Figure 1). This review highlights that survival and virulence depend on highly specialized protein machinery. The characterization of the structural proteome of the trophozoite cytoskeleton (Figure 2) – particularly the giardin family and tubulins with their diverse PTMs – reveals that the ventral disk is a sophisticated and unique attachment apparatus. This structure represents an ideal target for disrupting parasite colonization; similarly, the flagella, which are essential for parasite motility, are proposed as strategic targets to limit displacement within the intestinal environment.

Furthermore, the expansion of the ‘pathoproteome’ concept highlights the crucial role of the secretome and EVs (Figure 2), which, for a long time, did not receive adequate attention until their diverse functions in the communication and pathogenicity of various parasites were identified. The characterization of proteases like Giardipain-1, the role of tenascins and VSP-mediated antigenic variation provides a roadmap for understanding epithelial barrier disruption and immune evasion. Simultaneously, these molecules represent underexplored targets for inhibition or blockade via novel drugs or vaccines; moreover, VSPs are proposed as key proteins for immunodiagnostics (Figures 2 and 3).

While most current anti-giardial drugs focus on inhibiting DNA synthesis or disrupting cytoskeletal assembly (Table 1), there is a compelling rationale to explore the inhibition of parasite metabolism through therapeutic targets such as GlTIM, G3PD, CK and GlFBPA (Table 2). On the other hand, the proteomic transitions during encystation, involving CWPs (CWP 1-3) and molecular chaperones (Hsp70/90), remain fundamental for intercepting the parasite’s transmission cycle, including the use of drugs that disrupt proper cyst formation (Table 2). In contrast to the trophozoite stage, toward which most therapies (Tables 1 and 2) and vaccine candidates (Table 3) are directed, the cysts and their wall proteins have served as the most accessible and widely used diagnostic targets for identifying infected individuals (Figure 3).

Regarding prophylaxis, the high prevalence of infection and rising drug resistance necessitate the development of effective vaccines. This review proposes a shift toward bivalent subunit vaccines that include both trophozoite (e.g. α1-giardin, VSPs) and cyst antigens (e.g. CWP2) to simultaneously target colonization and transmission. However, achieving cross-protection between assemblages A and B remains a significant challenge. The future of Giardia immunization lies in the integration of next-generation platforms, such as cationic lipid nanoparticles and mRNA-based vaccines, combined with mucosal adjuvants like chitosan to ensure antigen stability through the gastrointestinal tract.

Future research should prioritize the validation of pharmacological protein targets through functional studies and both *in vitro* and *in vivo* assays. In the clinical setting, there is an urgent need to shift from empirical treatments toward point-of-care diagnostics utilizing the novel antigens described herein. Finally, the integration of biomaterials and multi-antigenic platforms in vaccine development – leveraging the immunogenic potential of the surface proteome – is essential to counteract the rising reports of drug resistance and, ultimately, to reduce the global burden of giardiasis.

## Figure 1 Long description

The diagram illustrates the life cycle of Giardia with numbered steps: 1) Ingestion of food or water contaminated with mature cysts. 2) Excystation and release of trophozoites in the small intestine. 3) Multiplication of trophozoites. 4a) Excretion of cysts in feces. 4b) Patients with diarrhea may excrete trophozoites. 5) Arthropods can transport cysts from feces to food. 6) Feces from infected animals can contaminate food and water sources. 7) Food and water contaminated with viable cysts capable of infecting humans. The diagram includes images of a human digestive system, trophozoites, feces, a dog, food and an arthropod, illustrating the transmission and infection process.

Navigate back to Figure 1.

## Figure 2 Long description

The image A shows a structural schematic of a trophozoite, illustrating various components labeled with letters. At the top left, nuclei are marked as N, surrounded by flagella labeled F, which extend from the basal bodies labeled Bb. The median body, marked Mb, is positioned centrally, while the ventral disk labeled Vd is located below it. The axoneme, marked Ax, runs vertically through the center. Encystation-specific vesicles, labeled ESVs, are scattered throughout the structure, with peripheral vesicles marked Pv located towards the bottom. Extracellular vesicles, labeled EVs, are shown on the right side. The image B represents major trophozoite proteins and their subcellular localization, with corresponding proteins listed on the right. Tubulins are depicted in green, giardins in blue, microtubule-associated proteins in pink, cysteine proteases in orange, variant-specific surface proteins in yellow and tenascins in purple. Nitroreductase and pyruvate-ferredoxin oxidoreductase are shown in red, NADH oxidase in dark blue, ribosomal proteins in gray, fructose-1,6-biphosphate aldolase in light purple, glycerol-3-phosphate dehydrogenase in brown, triose phosphate isomerase enzyme in dark purple, arginine deiminase in light blue, cyst wall synthase in dark purple, striated fibre assemblin-like protein in light purple, heat shock proteins in dark purple and head-stalk proteins in light purple. Diagnostic targets are indicated with a microscope symbol, drug targets with a capsule symbol and vaccine targets with a syringe symbol.

Navigate back to Figure 2.

## Figure 3 Long description

The image consists of two parts labeled A and B. The image A shows a structural schematic of a cyst. On the left side, various components are labeled: CW for cyst wall, N for nuclei, Ax for axoneme, Df for ventral disk fragments and V for vesicles. The cyst wall is depicted as a multi-layered structure surrounding the cyst. The nuclei are shown as circular structures within the cyst. The axoneme is represented as elongated structures, while ventral disk fragments and vesicles are scattered throughout the cyst. The image B illustrates the representation of major cyst proteins and their subcellular localization. It includes CWPs for cyst wall proteins, HCNCp for high cysteine non-variant cyst protein, HSPs for heat shock proteins and gEno for enolase. Diagnostic targets and vaccine targets are indicated with specific symbols. The proteins are shown in different colors and shapes to distinguish their types and functions within the cyst structure.

Navigate back to Figure 3.

## Table 1 Long description

The table lists six antigiardial drug classes and links each to a primary proteomic target and its main cellular effect in Giardia. Nitroimidazoles target PFOR and ferredoxin and are activated into radicals that damage DNA and proteins. Benzimidazoles bind beta tubulin and block microtubule formation, disrupting cytoskeletal structure and motility. Nitazoxanide is associated with NRT-1, PFOR, and quinone reductase and is described as interfering with energy metabolism. Paromomycin targets ribosomal proteins and inhibits protein synthesis by causing erroneous mRNA reading. Furazolidone targets NADH oxidase, generating superoxide radicals and inhibiting encystation, while chloroquine targets ventral disc and adhesion proteins to disrupt intestinal attachment and colonization. Targets span metabolism, genetic and protein machinery, and attachment structures, but the table summarizes proposed mechanisms and does not compare dose, efficacy, or resistance.

Navigate back to Table 1.

## Table 2 Long description

The table lists experimental and proposed therapeutic candidates for giardiasis, pairing each agent class with its Giardia molecular target, affected pathway, selectivity rationale, and supporting citations. Several approaches disrupt parasite energy metabolism, including RNA interference against arginine deiminase and carbamate kinase modulators that block ATP production from arginine; carbamate kinase is noted as absent from the human genome. Two glycolysis strategies focus on enzymes that differ from human counterparts: inhibitors of Giardia class two fructose bisphosphate aldolase and inhibitors of Giardia triose phosphate isomerase aimed at a cysteine residue unique to the parasite. Stage transition and transmission are addressed by heat shock protein ninety inhibition, which alters encystation, and by phosphonoxins that inhibit cyst wall synthase and prevent formation of the GalNAc-based cyst wall lattice. NBDHEX targets glycerol three phosphate dehydrogenase and disrupts redox balance, with potential activity against thioredoxin reductase and reported higher in vitro efficacy than metronidazole. Overall, selectivity is mainly justified by targets being absent in humans, structurally divergent from human enzymes, or containing parasite-unique residues. The entries summarize proposed mechanisms and rationales rather than providing comparable dosing, safety, or clinical effectiveness outcomes.

Navigate back to Table 2.

## Table 3 Long description

The table compiles experimental vaccine candidates against Giardia, listing the parasite species or strain, antigen, formulation, administration route, immune readouts, and reported protection or safety, with citations. Early whole-trophozoite immunizations in mice and dogs often increased antibodies and, when challenged, reduced cyst output or intestinal trophozoite burden; one mouse study reported most immunized animals stopped passing fecal cysts for eight weeks. Several oral vaccines targeting cyst wall protein 2, delivered with mucosal adjuvants or live bacterial vectors, repeatedly showed fewer cysts shed after challenge, commonly around two thirds to nine tenths lower than controls, and sometimes increased gut IgA and T cell or B cell markers in lymphoid tissues. A licensed killed-trophozoite dog vaccine was associated with symptom resolution and eventual cessation of cyst shedding, with no adverse reactions reported in that study. Other antigens such as variant surface protein mixtures, giardins, and selected enzymes generally raised serum IgG and fecal IgA and sometimes reduced trophozoites, while some entries report immune responses only and did not assess protection. Comparisons across rows are limited because challenge models, host species, outcome measures, and follow-up times differ, and several studies report qualitative outcomes rather than standardized efficacy endpoints.

Navigate back to Table 3.
